# Prohibitin Expression Deregulation in Gastric Cancer Is Associated with the 3′ Untranslated Region 1630 C>T Polymorphism and Copy Number Variation

**DOI:** 10.1371/journal.pone.0098583

**Published:** 2014-05-30

**Authors:** Mariana Ferreira Leal, Priscila Daniele Ramos Cirilo, Tatiane Katsue Furuya Mazzotti, Danielle Queiroz Calcagno, Fernanda Wisnieski, Samia Demachki, Margarita Cortes Martinez, Paulo Pimentel Assumpção, Roger Chammas, Rommel Rodríguez Burbano, Marília Cardoso Smith

**Affiliations:** 1 Disciplina de Genética, Departamento de Morfologia e Genética, Universidade Federal de São Paulo, São Paulo, SP, Brazil; 2 Departamento de Ortopedia e Traumatologia, Universidade Federal de São Paulo, São Paulo, SP, Brazil; 3 Laboratório de Oncologia Experimental, Departamento de Radiologia, Faculdade de Medicina, Universidade de São Paulo, São Paulo, SP, Brazil; 4 Centro de Investigação Translacional em Oncologia, Instituto do Câncer do Estado de São Paulo, São Paulo, SP, Brazil; 5 Núcleo de Pesquisa em Oncologia, Hospital Universitário João de Barros Barreto, Universidade Federal do Pará, Belém, PA, Brazil; 6 Laboratório de Citogenética Humana, Instituto de Ciências Biológicas, Universidade Federal do Pará, Belém, PA, Brazil; Ohio State University Medical Center, United States of America

## Abstract

PHB is a reported oncogene and tumor suppressor in gastric cancer. Here, we evaluated whether the *PHB* copy number and the rs6917 polymorphism affect its expression in gastric cancer. Down-regulation and up-regulation of *PHB* were observed in the evaluated tumors. Reduced expression was associated with tumor dedifferentiation and cancer initiation. The T allele of the rs6917 polymorphism was associated with reduced *PHB* mRNA levels. Moreover, the up-regulation of *PHB* appeared to be regulated by the gain of additional gene copies. Thus, *PHB* copy number variation and differential expression of the rs6917 polymorphism may play a role in *PHB* transcriptional regulation.

## Introduction

Gastric cancer (GC) is the fourth most common cancer and the second leading cause of cancer-related deaths worldwide [1]. Despite significant advances in the study of GC, the molecular alterations involved in gastric carcinogenesis remain unknown.

Chromosome 17 is one of the most common chromosomes exhibiting numerical aberrations in GC (see review [2]). Our group has previously reported the presence of chromosome 17 aneuploidy, both gains and losses, in GC in individuals in Northern Brazil [3], [4], [5], [6] and in all GC cell lines established from neoplasias in this population [7], [8]. Therefore, this chromosome may contain important genes involved in gastric carcinogenesis.

The prohibitin-1 (*PHB*) gene maps to the chromosome 17q21 locus. This gene encodes a ubiquitous, evolutionarily conserved protein that is found in a wide range of organisms, including bacteria, plants, yeast, protozoans and mammals [9]. PHB was originally thought to play a central role in the inhibition of cell-cycle progression. More recently, PHB has been characterized as a chaperone involved in the stabilization of mitochondrial proteins; thus, PHB has been implicated in several cellular processes, including the regulation of proliferation, apoptosis and gene transcription [10].

PHB appears to play a role in the development of different types of cancer. Overexpression of PHB has been reported in cancer of the cervix, esophagus, breast, lung, bladder, thyroid, ovary and prostate. In contrast, reduced PHB expression has been observed in gliomas, and somatic mutations in *PHB* have been detected in sporadic breast cancers [9]. In addition, a functional single nucleotide polymorphism (SNP) in the *PHB* gene, changing a cytosine to a thymine at position 1630 in the 3′ UTR (rs6917), creates a variant that lacks antiproliferative activity [11], [12] and subsequently may increase the risk of malignant growth. The T allele of this SNP has been associated with an increased risk of breast cancer [13] and melanoma [14]. Therefore, the role of PHB in cancer proliferation and/or suppression remains controversial.

The role of PHB in gastric carcinogenesis has not been fully elucidated. Some previous studies have described increased PHB expression in GC samples [15], [16], [17], [18] and the serum of GC patients [19]. However, other studies have reported PHB down-regulation in this type of cancer [20], [21]. The investigation of the molecular mechanisms involved in the transcriptional regulation of *PHB* may provide new insights into its role in GC and aid the development of new anticancer treatments.

In this study, we first evaluated the mRNA and protein expression of PHB in GC and matched non-neoplastic gastric tissue samples. In addition, the possible associations between *PHB* and clinicopathological characteristics were investigated. Because the *PHB* gene is located in a chromosomal region frequently involved in numerical aberrations in GC [2], we also evaluated the *PHB* copy number in the tumor samples. Moreover, the allele-specific expression of *PHB* mRNA was assessed to investigate the relative transcription of each allele in heterozygous subjects with the rs6917 polymorphism as a possible mechanism involved in *PHB* regulation. To our knowledge, no previous study has evaluated *PHB* copy number and allele-specific expression in tumor samples.

## Materials and Methods

### Tissue Samples

Forty-eight pairs of GC samples and corresponding non-neoplastic gastric tissue samples (>5 cm from the edge of the tumor) were used to evaluate *PHB* mRNA expression and the SNP rs6917 genotype. In 38 of these GC samples, the *PHB* copy number was also assessed. PHB immunoreactivity was evaluated in 12 GC specimens.

All of the gastric samples were obtained from patients who underwent gastrectomy for GC at João de Barros Barreto University Hospital (HUJBB) in Northern Brazil. All of the patients had negative histories of exposure to either chemotherapy or radiotherapy before surgery, and there was no co-occurrence of other diagnosed cancers. Written informed consent with approval of the ethics committee of HUJBB was obtained.

Part of each dissected tumor sample was formalin-fixed and paraffin embedded (FFPE). Sections of FFPE tissue were stained with hematoxylin-eosin for histological evaluation or used for immunohistochemistry (IHC) analysis. Additional portions of each tumor and paired non-neoplastic tissue specimen were snap frozen in liquid nitrogen and stored at −80°C until nucleic acid purification.

All of the samples were classified according to Laurén [22], and the tumors were staged according to the TNM staging criteria [23].

### DNA and mRNA Purification

Total DNA and mRNA were simultaneously isolated from gastric tissue samples using an AllPrep DNA/RNA/Protein Kit (Qiagen, Germany) according to the manufacturer’s instructions. The DNA and RNA concentrations and quality were determined using a NanoDrop spectrophotometer (Kisker, Germany), and the RNA integrity was determined by gel electrophoresis.

### PHB Gene Expression

*PHB* expression was evaluated by reverse transcription quantitative polymerase chain reaction (RT-qPCR). First, complementary DNA (cDNA) was synthesized using a High-Capacity cDNA Archive kit (Applied Biosystems, Poland) according to the manufacturer’s protocol. Real-time RT-qPCR analysis was performed using QuantiFast SYBR Green Master Mix (Qiagen, German) on a 7500 Fast Real-Time PCR machine (Applied Biosystems, USA). The following primers (150 nM each) were designed for RT-qPCR amplification: *PHB* F5′-GTGTGGTTGGGGAATTCATGTGG-3′ and R5′-CAGGCCAAACTTGCCAATGGAC-3′; *ACTB* F5′-AGAAAATCTGGCACCACA-3′ and R5′-AGAGGCGTACAGGGATAG-3′. All of the reactions were performed in triplicate for both the target gene and the internal control (*ACTB*). *PHB* expression was normalized to *ACTB* expression. The abundance of mRNA expression was adjusted by amplification efficiency (*PHB* = 99%, *ACTB* = 102%) [24]. A non-neoplastic gastric tissue sample was designated as a calibrator for each paired tumor to calculate the relative quantification (RQ) [24].

### PHB Protein Expression

The paraffin sections were subjected to IHC. Tumor tissue sections (3 or 4 mm thick) were deparaffinized in xylene and rehydrated in a graded ethanol series. Epitope retrieval was performed in citrate buffer, pH 6.0, in humid heat in a pressure cooker. Next, the tissue sections were incubated with a primary mouse monoclonal antibody against PHB (II-14-10, dilution 1∶100; ThermoFisher Scientific, USA). Sites of immunoreactivity were visualized using a SuperPicture Polymer detection kit (Invitrogen, USA). The slides were viewed by light microscopy using a Nikon Eclipse E600 microscope (Nikon, USA) equipped with a digital Nikon DSM1200F camera (Nikon, USA). The non-stained region (white region) was selected and set as the background. Any staining was considered to be a positive result, regardless of the intensity. Negative controls, in which the primary antibody was replaced by 5% bovine serum albumin (BSA) in phosphate-buffered saline (PBS), were included in all series, and sections of normal human prostate tissue were used as positive controls.

### *PHB* Copy Number

To evaluate copy number variations (CNV), the qPCR procedure was performed using quantitative TaqMan CNV assays (Life Technologies, USA) for the *PHB* gene (Hs00178432_cn) and for the internal control *RNAse P* (#4403326). Multiplex qPCR reactions were performed in quadruplicate with gDNA according to the manufacturer’s protocol and cycling conditions in a 7500 Fast Real-Time PCR machine (Life Technologies, USA). The relative copy number of each sample was estimated using Copy Caller Software V1.0 (Life Technologies, USA). Commercial human gDNAs (G1471 and G1521; Promega, USA) were used for calibration.

### *PHB* Genotyping

DNA from non-neoplastic samples was used for *PHB* genotyping. The subjects were genotyped for the rs6917 polymorphism using Custom TaqMan SNP probes and primers (Applied Biosystems, Foster City, CA). The following primers and MGB probes were designed for allelic discrimination: primers F5′-TTGGTCCCTCTCAGATACCCA-3′ and R5′-CCGTGAGAAGGGCAGTCTCT-3′; FAM-labeled probe 5′-CTGCCAAAGA**C**GTGT-3′; and minor allele VIC-labeled probe 5′-CTGCCAAAGA**T**GTGT-3′.

### *PHB* Allele-specific Expression

Allele-specific expression was first determined by sequencing. Twenty to 40 ng of DNA or cDNA was used as a template for PCR amplification with the primers used for *PHB* genotyping. Before sequencing, the PCR products were separated using 2% agarose gel electrophoresis, and the specific band was extracted and purified. Sequencing was performed using the forward primer used for PCR amplification and a BigDye Terminator v3.1 Cycle Sequencing kit (Applied Biosystems, USA). Sequencing products were separated using an ABI 3500 Genetic Analyzer (Applied Biosystems, USA). Some of the samples were analyzed at least twice, including distinct RT-PCRs and sequencing assays.

The allelic expression levels were determined using PeakPicker software [25]. The software was used to first perform a normalization step, in which the SNP allele height was compared with the height of reference peaks in flanking sequences. We limited this normalization step to within a 21-base window. Ratio values above 1 were transformed to 1/(ratio) to transform all of the values to a 0–1 scale, and the values were then adjusted to the mean of the peak intensity ratios from a reference DNA sample heterozygous for the rs6917 polymorphism. Genomic DNA from heterozygous (N = 3) and homozygous samples (N = 3, for genotype CC; N = 2, for genotype TT) was used to validate the analysis and to confirm the allelic ratios of 50∶50 and 0∶100, respectively. In addition, cDNA from tissue samples (tumoral and non-tumoral specimens) from the two subjects homozygous for the minor allele in the rs6917 polymorphism were also used as controls.

**Figure 1:**
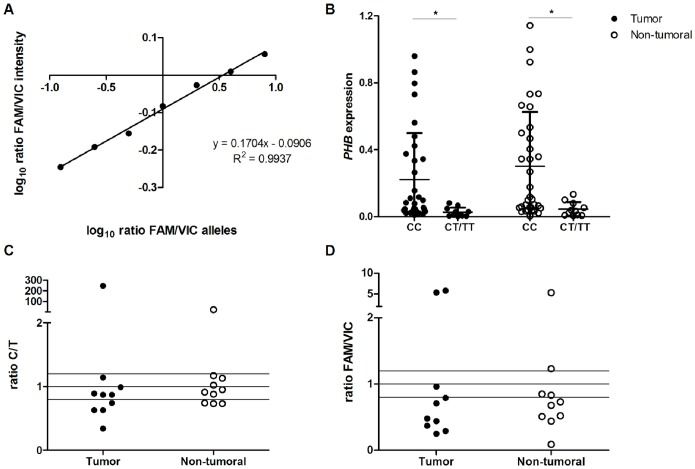
rs6917 allele-specific *PHB* expression. A) The log_10_ of FAM/VIC intensity ratio for *PHB* plotted against the log_10_ of FAM/VIC allele ratio of mixing homozygous DNAs at different ratios (8∶1, 4∶1, 2∶1, 1∶1, 1∶2, 1∶4, 1∶8 FAM/VIC allele). B) *PHB* expression by rs6917 genotype in gastric samples. C) C/T allelic ratio in cDNA from gastric samples of heterozygous patients by sequencing; D) FAM/VIC allelic ratio in gastric samples of heterozygous patients using a Custom Genotyping TaqMan assay, in which a specific probe for the C allele was labeled with FAM dye and a minor allele T probe was labeled with VIC dye. *Differentially expressed between groups by the T-test for independent samples, *P*<0.05.

Allele-specific *PHB* expression was also evaluated in heterozygous samples by RT-qPCR, as previously described [26]. Using a Custom TaqMan SNP genotyping assay for *PHB* genotyping, we generated a linear regression curve for the log10 fluorescence intensity *vs* the log10 allelic ratio based on the serial dilution of CC- and TT-genotyped genomic DNAs from control samples (samples from two homozygous individuals) in several ratios (CC:TT 8∶1, 4∶1, 2∶1, 1∶1, 1∶2, 1∶4, 1∶8) by RT-qPCR (Figure 1A). The allelic ratio of gene expression was extrapolated by the intercept of the log10 of FAM:VIC intensity on the standard curve. ROX (internal reference dye) and non-specific FAM and VIC fluorescence was normalized in all analyses. All of the reactions were performed in duplicate.

For the heterozygous cDNA samples, allelic ratios <0.8 or >1.2 were considered indicative of allele-specific expression.

### Statistical Analysis

For mRNA expression analysis, we first assessed the assumption that the data had a normal distribution using the Shapiro-Wilk normality test to determine the appropriate tests for subsequent statistical comparisons. The *PHB* mRNA levels were not normally distributed and were transformed (z-score) for analysis. Observations >+2 or <–2 were considered outliers and excluded from the analyses. A paired t-test was performed to compare the mean *PHB* expression between non-neoplastic and tumor samples. The T-test for independent samples and one-way ANOVA followed by the Games-Howell post-hoc test were used to evaluate the possible associations between *PHB* expression and clinicopathological characteristics, protein immunoreactivity, gene copy number and genotype. The Chi-square test or Fisher’s exact test was used to assess the relationship between *PHB* copy number and immunoreactivity and clinicopathological factors. Pearson’s correlation was used to evaluate a possible correlation between sequencing and the TaqMan assay for allele-specific expression analysis. In all of the analyses, *P*<0.05 was considered significant.

## Results

### *PHB* mRNA Expression in Gastric Tumors

*PHB* expression did not differ between neoplastic and matched non-neoplastic gastric samples (0.173±0.255 *vs* 0.227±0.297, *P* = 0.149). However, the mRNA levels were reduced at least 1.5-fold in 20 (45.5%) of the GC samples and increased in 9 (20.5%) when compared with paired non-neoplastic gastric tissue samples.

Table 1 shows the associations between *PHB* expression and the clinicopathological characteristics as well as the protein immunoreactivity, rs6917 genotype and gene copy number. Poorly differentiated tumors presented reduced *PHB* expression when compared with moderately differentiated tumors (*P* = 0.029; Table 1). However, both poorly differentiated GC (0.025±0.022 *vs* 0.239±0.303; *P*<0.001, ANOVA followed by the Games-Howell post-hoc test) and moderately differentiated GC (0.057±0.051 *vs* 0.239±0.303; *P*<0.001, ANOVA followed by the Games-Howell post-hoc test) presented reduced *PHB* expression compared with non-neoplastic gastric tissue samples.

**Table 1:** Clinicopathological characteristics and *PHB* expression in gastric cancer samples.

Variable (N)^a^	*PHB* expression (Mean ± SD)	*P* value
**Gender**		
Male (28)	0.205±0.288	0.269
Female (16)	0.116±0.177	
**Onset (years)**		
<45 (8)	0.137±0.268	0.662
≥45 (36)	0.181±0.255	
**Tumor location**		
Cardia (6)	0.312±0.361	0.153
Non-cardia (38)	0.151±0.233	
**Histological subtype** b		
Diffuse-type (17)	0.271±0.310	0.068
Intestinal-type (27)	0.111±0.196	
**Differentiation**		
Moderately differentiated (12)	0.051±0.028	0.029 d
Poorly differentiated (10)	0.025±0.022	
**Stage** c		
Early (5)	0.044±0.027	0.002^d^
Advanced (39)	0.189±0.267	
**Tumor invasion** c		
T1/T2 (11)	0.046±0.028	0.002^d^
T3/T4 (33)	0.215±0.283	
**Lymph node metastasis** c		
Absent (10)	0.076±0.109	0.040^d^
Present (34)	0.201±0.279	
**Distant metastasis** c		
Unknown/absent (36)	0.154±0.239	0.302
Present (8)	0.258±0.320	
**PHB immunoreactivity**		
<80% of cells (5)	0.031±0.0208	0.036^d^
≥80% of cells (5)	0.0688±0.026	
***PHB*** ** copy number**		
Without gain (25)	0.160±0.271	0.298
With gain (10)	0.258±0.180	
**rs6917 genotype**		
CC (33)	0.222±0.278	<0.001^d^
CT or TT (11)	0.026±0.028	

N: number of individuals; SD: standard deviation.

a Number of samples after outlier exclusion;

b According to Laurén [22];

c According to TNM staging [23];

d Differentially expressed between groups by T-test for independent samples, *P*<0.05.

Reduced *PHB* expression was associated with lower invasion (*P* = 0.002; Table 1) and an absence of lymph node metastasis (*P* = 0.040; Table 1). In addition, early GC presented reduced *PHB* expression compared with advanced GC (*P* = 0.002; Table 1). Moreover, only early GC presented a significant reduction in *PHB* mRNA levels compared with the non-neoplastic samples (0.044±0.027 *vs* 0.239±0.303, *P*<0.001, ANOVA followed by the Games-Howell post-hoc test).

### PHB Immunoreactivity in Gastric Tumors

**Figure 2:**
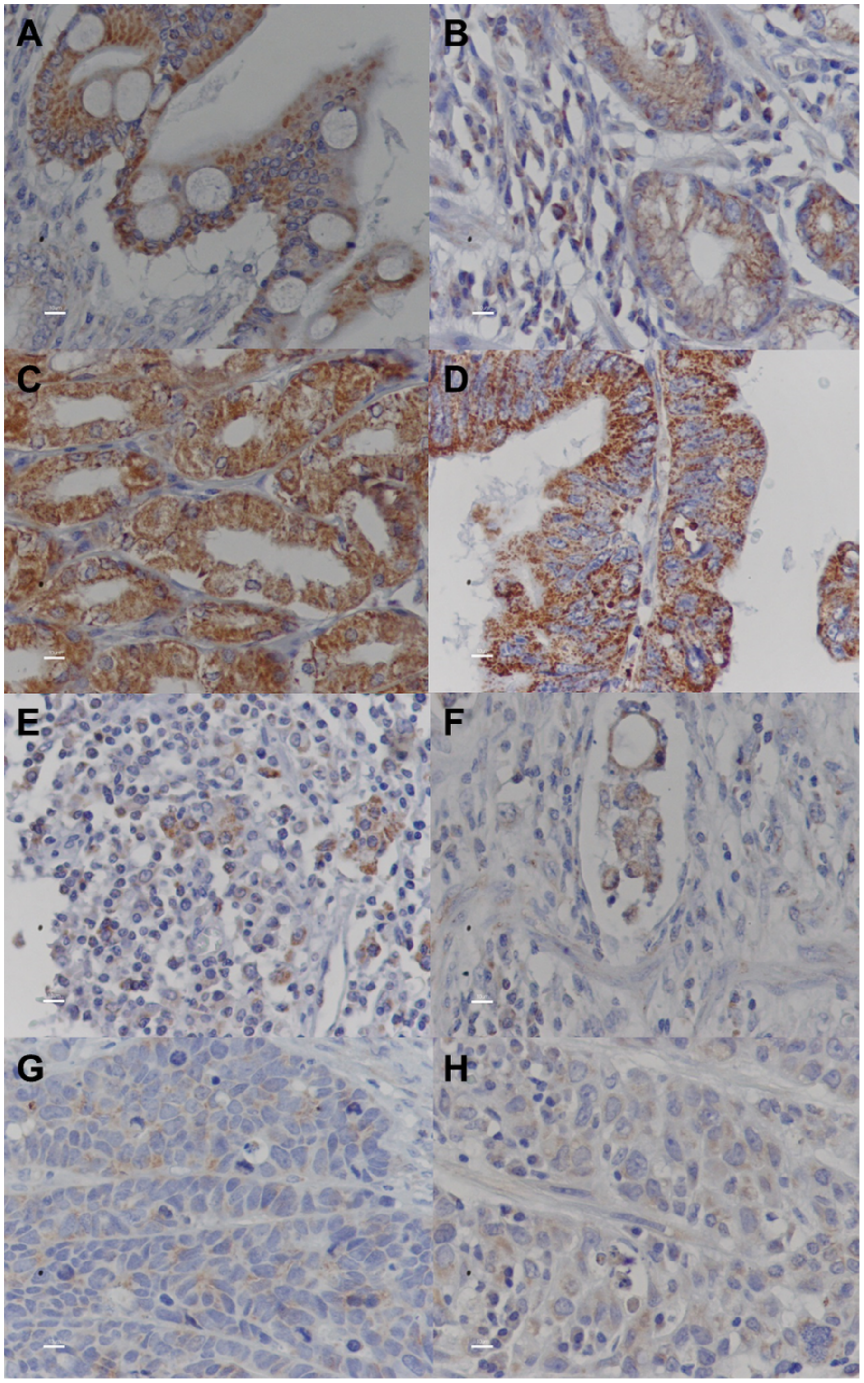
Immunohistochemical analysis of PHB in gastric samples. A) PHB staining in normal gastric mucosa (400x); B) PHB immunoreactivity in normal gastric mucosa and inflammatory cells (400x); C) strong PHB staining in intestinal metaplastic cells (400x); D) strong PHB staining in an intestinal-type tumor; E) moderate to intense PHB immunoreactivity in a poorly differentiated tumor; F) moderate to intense PHB immunoreactivity in a moderately differentiated tumor; G) weak PHB staining in a moderately differentiated tumor (400x); D) weak PHB staining in a diffuse-type tumor (400x).

Protein immunostaining was observed in all of the tumor samples evaluated by IHC (Table 2). In all cases, PHB immunoreactivity was detected in neoplastic and non-neoplastic cells, including intestinal metaplastic and inflammatory cells (Figure 2A–H). PHB was primarily expressed in the cytoplasm. The staining intensity and the percentage of immunoreactive cells varied among the studied cases (Table 2). Samples presenting <80% of tumor cells with PHB immunoreactivity showed reduced mRNA expression (*P* = 0.036, Table 1).

**Table 2:** Immunohistochemistry analysis in gastric tumors.

Case	Histological subtype^a^	Differentiation	Stage^b^	% of stained tumor cells	Intensity of staining
1	Intestinal-type	Moderately differentiated	1	80%	Moderate to strong
2	Intestinal-type	Poorly differentiated	4	70%	Weak and moderate
3	Intestinal-type	Moderately differentiated	4	60%	Moderate
4	Intestinal-type	Moderately differentiated	1	30%	Weak
5	Intestinal-type	Moderately differentiated	1	80%	Strong
6	Intestinal-type	Poorly differentiated	3	80%	Moderate to strong
7	Intestinal-type	Moderately differentiated	1	20%	Weak and Moderate
8	Intestinal-type	Moderately differentiated	3	90%	Strong
9	Intestinal-type	Moderately differentiated	4	70%	Moderate to strong
10	Intestinal-type	Moderately differentiated	2	80%	Moderate
11	Intestinal-type	Poorly differentiated	3	20%	Strong
12	Diffuse-type	Not applied	3	20%	Weak to strong

a According to Laurén [22];

b According to TNM staging [23].

### *PHB* Copy Number in Gastric Tumors

**Figure 3:**
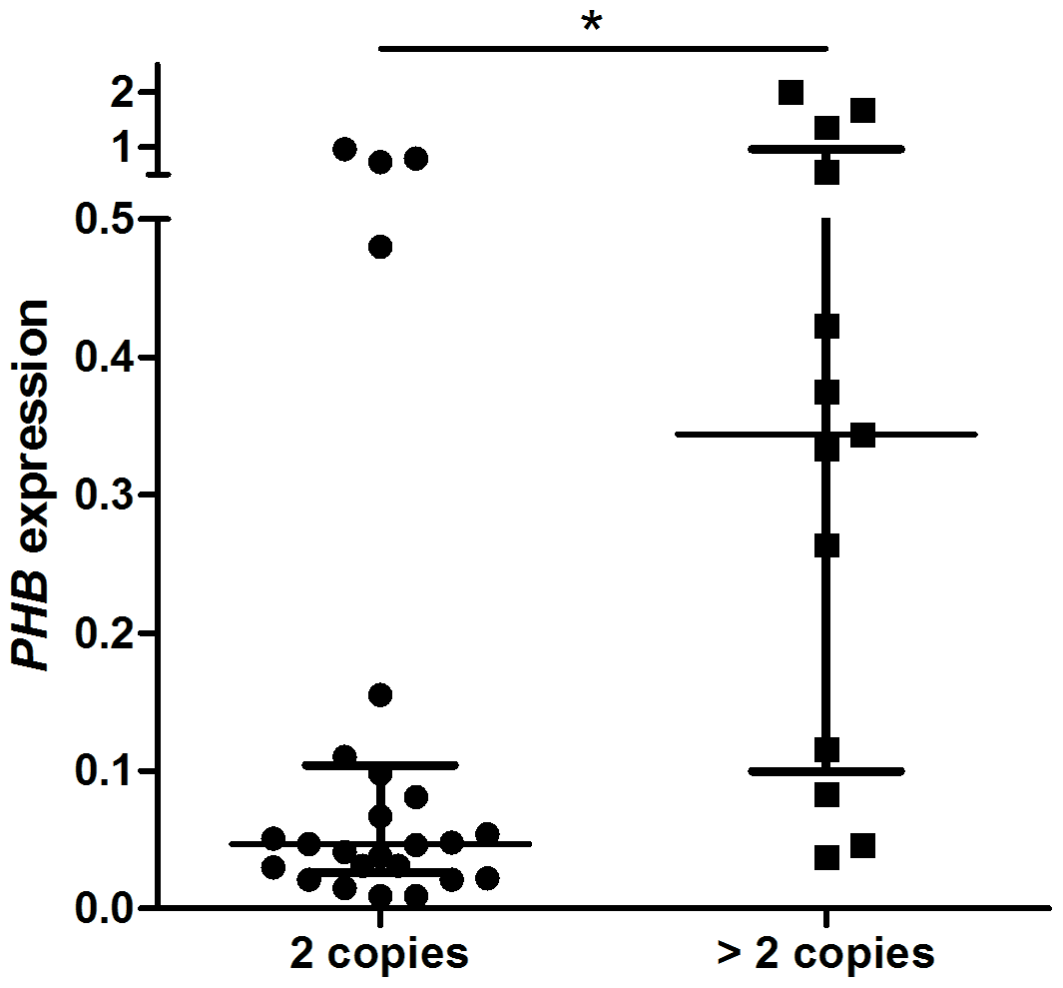
*PHB* mRNA expression by copy number. Lines show the median and interquartile range of *PHB* expression. *Differentially expressed between groups by the Mann-Whitney test, *P*<0.05.

*PHB* was amplified in 13 of 38 (34.2%) tumors, including 2 samples with 4 copies. No tumor presented a *PHB* deletion. Interestingly, 3 samples that presented outlier values for *PHB* mRNA expression also presented gene amplification. Therefore, when outlier values were included in the analysis, *PHB* expression was higher in samples with gene amplification than in those without gene amplification (median ± interquartile range: 0.344±0.335 *vs* 0.047±0.07; *P* = 0.003, non-parametric Mann-Whitney test; Figure 3). *PHB* gain was associated with late-onset GC compared with early-onset GC (*P* = 0.022; Table 2). No association between *PHB* copy number and protein immunoreactivity or any other clinicopathological characteristics was found (Table 3).

**Table 3:** Clinicopathological characteristics and *PHB* copy number in gastric cancer samples.

Variable (N)	*PHB* copies [N (%)]		*P* value
	2 copies	≥3 copies	
**Gender**			
Male (23)	15 (65.2)	8 (34.8)	0.604
Female (15)	10 (66.7)	5 (33.3)	
**Onset (years)**			
<45 (8)	8 (100)	0 (0)	0.022^c^
≥45 (30)	17 (56.7)	13 (43.3)	
**Tumor location**			
Cardia (7)	4 (57.1)	3 (42.9)	0.451
Non-cardia (31)	21 (67.7)	10 (32.3)	
**Histological subtype** a			
Diffuse-type (14)	9 (64.3)	5 (35.7)	0.577
Intestinal-type (24)	16 (66.7)	8 (33.3)	
**Differentiation**			
Moderately differentiated (10)	7 (70)	3 (30)	0.214
Poorly differentiated (6)	6 (100)	0 (0)	
**Stage** b			
Early (5)	4 (80)	1 (20)	0.433
Advanced (33)	21 (63.6)	12 (36.4)	
**Tumor invasion** b			
T1/T2 (10)	8 (80)	2 (20)	0.242
T3/T4 (28)	17 (60.7)	11 (39.3)	
**Lymph node metastasis** b			
Absent (9)	6 (66.7)	3 (33.3)	0.640
Present (29)	19 (65.5)	10 (34.5)	
**Distant metastasis** b			
Unknown/absent (29)	20 (69)	9 (31)	0.360
Present (9)	5 (55.6)	4 (44.4)	
**PHB immunoreactivity**			
<80% of cells (7)	7 (100)	0 (0)	0.416
≥80% of cells (5)	4 (80)	1 (20)	

N: number of individuals;

a According to Laurén [22];

b According to TNM staging [23];

c Differentially expressed between groups by Chi-square test or Fisher’s exact test, *P*<0.05.

### *PHB* Allele-specific Expression

We also investigated whether the presence of the minor allele in rs6917 was associated with the gene expression deregulation in GC patients. In our sample, 11 of 48 patients (22.9%) were heterozygous and 2 patients (4.17%) were homozygous for the minor allele in the rs6917 polymorphism. All of the samples with the T allele presented 2 copies of the *PHB* gene. The presence of the T allele in the rs6917 polymorphism was associated with reduced *PHB* expression in GC (*P*<0.001; Table 1; Figure 1B) and in non-neoplastic samples (mean ± SD: 0.302±0.325 *vs* 0.045±0.043; *P*<0.001; Figure 1B).

One pair of heterozygous samples (tumor and non-tumor) was not used for the analysis of allele-specific expression. A correlation between the allelic ratio of the rs6917 polymorphism determined by sequencing and the TaqMan assay was detected (*P* = 0.003; r = 0.627). By sequencing, approximately 50% of cases presented differential allelic expression (Figure 1C). However, the TaqMan assay showed that the T and C alleles presented differential allelic expression in most patients (Figure 1D). The degree of difference in the expression between the two alleles varied among individuals. The T to C allelic expression ratio was similar in most pairs of tumor and non-tumor samples. Only one patient presented a higher C/T ratio in both tumor and non-neoplastic samples in both assays. In most cases, a lower C/T ratio was detected regardless of the methodology applied (Figures 1C and 1D). No association between differential allelic expression and age of onset or any other clinicopathological variable was detected.

## Discussion

PHB appears to be essential in cellular homeostasis. Recent studies have suggested that PHB may act as a pro-tumorigenic and anti-tumorigenic factor in several cell types (see review [10]). In the present study, the protein and mRNA expression profiles of PHB presented a heterogeneous pattern among tumor samples. Although we did not find a significant difference between GC and matched non-neoplastic samples, we observed that the mRNA level was reduced 1.5-fold in 45.5% of GC samples and increased in 20.5% of tumors. The reduced mRNA expression was in part due to a reduced frequency of tumor cells presenting PHB immunoreactivity, which highlights the heterogeneity among tumor cell clones.

Liu et al. [21] described reduced mRNA expression in four gastric tumors compared with their corresponding normal tissues, which partly corroborates our results. Supporting an anti-tumorigenic role, PHB blocks entry into S phase [27]. In addition, the wild-type rs6917 *PHB* 3′UTR alone is able to inhibit cell cycle progression [11], [28] and tumor growth [12] as well as reduce cell mobility [29]. Furthermore, PHB plays a role in maintaining normal mitochondrial function and morphology [30]. It has been suggested that loss of mitochondrial PHB expression (cytoplasmic localization, as observed in our samples) could lead to accelerated proteolysis of membrane proteins and impair function of the mitochondrial respiratory chain [10]. Our previous proteomic study showed that several proteins involved in energy metabolism pathways (mitochondrial dysfunction, pyruvate metabolism, oxidative phosphorylation, citrate circle, glycolysis/gluconeogenesis) were deregulated [31] and suggested that prominent mitochondrial functions may be altered, shifting energy production in GC cells and suggesting the Warburg effect [32].

To our knowledge, few studies have evaluated the possible association between *PHB* mRNA levels and the rs6917 polymorphism. In one study, Tang et al. did not observe a significant association between the rs6917 polymorphism and mRNA expression in lymphoblastoid cell lines included in the HapMap database [33]. However, these authors reported that the T allele was associated with an increased risk of breast cancer. In the present study, we demonstrated that the presence of the T allele in the rs6917 polymorphism was associated with reduced *PHB* expression in non-neoplastic and neoplastic gastric cells. The rs6917 polymorphism is located in the 3′UTR and is present only in isoform 1 of PHB, which was detected in the present study by cDNA sequencing and a TaqMan assay. The 3′UTR contains multiple *cis*- and *trans*-elements, and these structures have a widespread influence on mRNA translation, stability and subcellular localization [34], [35], [36]. The T variant creates a potential binding site for the microRNAs has-miR-1292 and has-886-5p (http://snpinfo.niehs.nih.gov/cgi-bin/snpinfo/mirna.cgi?2_rs6917), which may alter gene expression by either mRNA decay or translation. Other microRNAs were previously associated with the regulation of *PHB* expression in GC. Liu et al. [21] have reported that *PHB* is regulated by miR-27a, which is commonly up-regulated in GC. The authors suggested that the down-regulation of *PHB* by this microRNA may explain why the suppression of miR-27a can inhibit GC cell growth. In addition, the 3′UTR of *PHB* encodes an antisense RNA (Gene ENSG00000250186; HAVANA http://www.sanger.ac.uk/). The presence of the rare allele in the antisense RNA may modify the secondary structure and decrease the kcal/mol when compared with the wide-type sequence using CLC RNA Workbench 4.0 software (data not shown). Therefore, the rs6917 polymorphism may lead to *PHB* down-regulation by creating new microRNA target sites or through its regulation by the antisense RNA in gastric cells, thus contributing to gastric carcinogenesis.

In our samples, most of the heterozygous patients presented increased expression of the T allele compared to the C allele. The difference in the relative mRNA levels of the two alleles could be the result of differences in transcription or mRNA stability, and they show that this polymorphism presents a *cis* effect in gastric cells. To our knowledge, this is the first study to evaluate the differential *PHB* allele-specific expression in tumor samples. Previous studies have demonstrated that a *PHB* variant with the T allele at position 1630 in the 3′UTR lacks anti-proliferative and tumor growth suppression abilities in vitro and in animal models [29]. Therefore, the presence of the T allele, particularly when it presents elevated expression relative to the C allele, may induce a “sponge effect” for post-transcriptional regulators such as miRNAs and contribute to gastric cell proliferation, predisposing heterozygous individuals to GC.

The possible effect of *PHB* down-regulation – due to the rs6917 polymorphism, for example – on GC risk is in agreement with the associations between reduced *PHB* mRNA and lower invasion, lack of lymph node metastasis and early-stage GC. To our knowledge, no previous study in the literature has evaluated the possible association between *PHB* expression and GC prognostic factors. These findings suggest that *PHB* down-regulation may be required for tumor initiation.

However, previous proteomic studies have reported PHB upregulation in gastric tumors [15], [16], [17]. Additionally, Kang et al. [18] have reported the overexpression of PHB protein and mRNA in GC (compared with adjacent normal gastric tissues) in Asian individuals. In our sample, 20.5% of tumors also presented increased *PHB* expression. PHB appears to play a role in cell proliferation, adhesion and migration and, therefore, in the progression of malignant transformation through RAS-RAF signaling [37]. We hypothesize that an increased *PHB* mRNA level is required for GC progression. The evaluation of human samples allows only the investigation of a single time point (at the time of surgical repair); thus, we are unable to evaluate the dynamic regulation of gene expression. However, *PHB* expression is most likely related to the cellular context and molecular background of gastric cells.

Kang et al. [18] have reported that PHB protein and mRNA expression differ between moderately and poorly differentiated GC and that only poorly differentiated tumors present PHB overexpression compared with non-neoplastic samples. In our sample, few tumors were classified according to the degree of differentiation. However, we observed that both poorly and moderately differentiated tumors presented reduced *PHB* expression compared with non-neoplastic samples and that the *PHB* mRNA levels were lower in the poorly differentiated tumors. Therefore, in our samples, *PHB* expression appeared to decrease with tumor dedifferentiation. Further investigations are necessary for a better understanding of PHB’s role in the differentiation process in normal and neoplastic gastric cells.

In this study, we observed that 34.2% of tumors gained copies of *PHB*. Alterations in chromosome 17 have been associated with tumor progression and malignant potential in primary GC [38], [39]. To our knowledge, this is the first study to use an accurate and robust technique to evaluate the *PHB* copy number in GC samples. We observed an association between *PHB* copy number and mRNA levels, revealing a cis-dosage effect of CNV on gene expression levels. Therefore, our results suggest that CNV is an important element in driving downstream *PHB* transcription in some gastric tumors. This genetic mechanism, mainly observed in late-onset GC, may contribute for the *PHB* role as oncogene. This finding also highlight that several mechanisms may lead to *PHB* deregulation in GC cells.

The increase in *PHB* copy number was associated with late-onset GC. Clinicopathological differences between early-onset and late-onset GC have been described [40], [41], [42], but little is known about the genetic changes associated with the age of onset of GC [2]. Buffart et al. [43] previously demonstrated that young and old patients belong to groups with different genomic profiles. The amplification of the *PHB* locus highlights the heterogeneity of GC.

The main limitation of this study is its relatively small sample size. Therefore, some statistical analysis presented reduced power to detect significant differences between groups probably due to the large heterogeneity among samples. Therefore, false-negative results may have occurred. Further evaluations are still necessary to evaluate the role of *PHB* in gastric carcinogenesis and its transcriptional regulation.

In conclusion, both the down-regulation and up-regulation of *PHB* may be observed in GC. The reduction of *PHB* expression appears to be involved in the tumor dedifferentiation process and cancer initiation. The T allele in the *PHB* rs6917 polymorphism may contribute to the observed reduction in *PHB* mRNA levels and therefore contribute to GC risk. However, *PHB* up-regulation appears to be regulated by gene copy number. Differential expression of the rs6917 polymorphism in gastric samples was reported for the first time, and this variation may play a role in the regulation of *PHB* expression. The pleiotropic function of *PHB* highlights the necessity of further investigations in GC because its activity in gastric cells is likely to be tightly regulated to avoid possible adverse consequences of decreased or increased expression.
